# Sigma-1 receptor expression in a subpopulation of lumbar spinal cord microglia in response to peripheral nerve injury

**DOI:** 10.1038/s41598-023-42063-8

**Published:** 2023-09-07

**Authors:** Ethan Schonfeld, Thomas Michael Johnstone, Ghani Haider, Aaryan Shah, Neelan Joseph Marianayagam, Sandip Biswal, Anand Veeravagu

**Affiliations:** 1 Neurosurgery Artificial Intelligence Lab, Stanford University School of Medicine, Stanford, CA USA; 2 Department of Neurosurgery, Stanford University School of Medicine, Stanford, CA USA; 3 Department of Radiology, Stanford University School of Medicine, Stanford, CA USA

**Keywords:** Computational biology and bioinformatics, High-throughput screening, Machine learning, Diagnostic markers

## Abstract

Sigma-1 Receptor has been shown to localize to sites of peripheral nerve injury and back pain. Radioligand probes have been developed to localize Sigma-1 Receptor and thus image pain source. However, in non-pain conditions, Sigma-1 Receptor expression has also been demonstrated in the central nervous system and dorsal root ganglion. This work aimed to study Sigma-1 Receptor expression in a microglial cell population in the lumbar spine following peripheral nerve injury. A publicly available transcriptomic dataset of 102,691 L4/5 mouse microglial cells from a sciatic-sural nerve spared nerve injury model and 93,027 age and sex matched cells from a sham model was used. At each of three time points—postoperative day 3, postoperative day 14, and postoperative month 5—gene expression data was recorded for both spared nerve injury and Sham cell groups. For all cells, 27,998 genes were sequenced. All cells were clustered into 12 distinct subclusters and gene set enrichment pathway analysis was performed. For both the spared nerve injury and Sham groups, Sigma-1 Receptor expression significantly decreased at each time point following surgery. At the 5-month postoperative time point, only one of twelve subclusters showed significantly increased Sigma-1 Receptor expression in spared nerve injury cells as compared to Sham cells (p = 0.0064). Pathway analysis of this cluster showed a significantly increased expression of the inflammatory response pathway in the spared nerve injury cells relative to Sham cells at the 5-month time point (p = 6.74e−05). A distinct subcluster of L4/5 microglia was identified which overexpress Sigma-1 Receptor following peripheral nerve injury consistent with neuropathic pain inflammatory response functioning. This indicates that upregulated Sigma-1 Receptor in the central nervous system characterizes post-acute peripheral nerve injury and may be further developed for clinical use in the differentiation between low back pain secondary to peripheral nerve injury and low back pain not associated with peripheral nerve injury in cases where the pain cannot be localized.

## Introduction

Low back pain (LBP) is the leading cause of years lived with disability worldwide^1^. Specific treatment (e.g.: epidural steroid injection, radiofrequency ablation, corrective surgery) is often limited as localization of the source is problematic. This results in frequent opioid prescriptions, worsening the opioid epidemic^2^. In some cases, structural abnormalities identified by conventional imaging may not be causally related to the LBP, such that surgical correction does not improve a patient’s pain symptoms. Conversely, in some cases there are no structural abnormalities found in conventual imaging, and thus specific therapies cannot be pursued. In an effort to better localize LBP, a radioligand that binds to the Sigma-1 Receptor (S1R), which is a molecular biomarker of nerve injury and neuroinflammation^3^, is used as a tracer for PET/MRI^4^. This pain localization has already been well developed, and found to identify pain sources both included and not included in structural imaging for chronic knee pain^4,5^, and in recent work, LBP^6,7^.

Despite these advances, S1R has not been fully characterized in its biological role and response to pain. It is clear that S1R localizes to local pain sources. The same S1R radiotracer was able to localize peripheral nerve injury (PNI) sites in a neuropathic pain model^8^. However, it is also reported that S1R is expressed in the central nervous system (CNS), with almost all neurons in the spinal cord expressing S1R, specifically those in the ventral horn motor neurons^9,10^. S1R expression is not selectively neuronal, as consistent with its role in pain, S1R is expressed in microglia as a neuroinflammatory modulator^11^. However, it is unclear how S1R expression in the spinal cord is affected by PNI. Preliminary work in a spinal nerve ligation model found downregulation of S1R in injured dorsal root ganglion neurons and their surrounding glial cells, with a lesser degree of downregulation in neighboring uninjured neurons^12^.

It is vital to understand the S1R response in non-neuronal cell types, specifically microglial as the drivers of neuroinflammation following PNI. This study hypothesized that spinal cord microglial subpopulations would differentially express S1R based on whether they were exposed to a PNI or sham surgery. If such an upregulated response was found, there would be an increase in clinically meaningful information in the S1R radioligand PET/MRI imaging method. A significant change in S1R expression in microglia exposed to PNI relative to those exposed to sham surgery may be clinically useful in differentiating LBP secondary to PNI from non-PNI LBP. Therefore, this study aimed to determine if any microglial subpopulation’s expression of S1R varied between sham surgery and PNI treatments at different postoperative time points. It also sought to identify the cellular pathways that were differentially regulated in these microglial subpopulations.

## Methods

### Data source

A publicly available transcriptomic dataset of 102,691 L4/5 mouse microglial cells from a sciatic-sural nerve spared nerve injury (SNI) model and 93,027 age and sex matched cells from a sham model was used^13^. Both SNI and Sham cell groups contained three conditions: Day 3, Day 14, and Month 5 post-surgery when the sample was collected. For all cells 27,998 genes were sequenced. Day 3 and 14 correspond to acute PNI timepoints, where inflammation is reduced at Day 14. The Month 5 timepoint corresponds to a chronic PNI state. This lumbar spinal microglia population was found to exhibit time and sex specific responses to PNI^13^.

### Data download

Single-cell RNA-sequencing data form the data source above was downloaded from the Gene Expression Omnibus under accession GSE162807. All count files were downloaded as 10× CellRanger hdf5 file type and were read into a Seurat object using R (version 4.2.1) with the Seurat package (version 4.3.0). As there were duplicates for some samples, one sample for each gender for each condition was chosen to continue in our workflow.

### Data preprocessing

According to the standard Seurat single-cell RNA-sequencing preprocessing pipeline, data was first normalized using a log normalization method. To duplicate the preprocessing of the data from Tansley et al., all cells with under 500 or over 4000 features were removed from the dataset^13^. 2000 variable features were selected with the ‘vst’ method, and using these variable features the data was scaled and principal component analysis (PCA) was performed.

### Clustering and visualization

The same pipeline to cluster the cells from Tansley et al. was performed^13^. Nearest neighbors for each cell were found using the first 15 dimensions, and the clusters of the cells were computed using a shared nearest neighbor approach with resolution parameter set to 0.3. 12 clusters of cell subpopulations were found. Visualization of the resulting clusters and data was performed using Uniform Manifold Approximation and Projection (UMAP).

### Pathway analysis

Using Seurat, a gene set enrichment ranking was computed, and the fgsea package (version 1.24.0) in R was used to run a gene set enrichment analysis. 50 pathways were included in the analysis from the Molecular Signatures Database Hallmark Pathway Set ("mh.all.v2022.1.Mm.symbols.gmt").

### Statistical analysis

A one-tailed t-test was utilized to assess significant differences in S1R expression between SNI and Sham cells. A one-tailed t-test was used as opposed to a two-tailed t-test as the current clinical imaging method specifically tests for and identifies an increase in S1R expression to localize pain generators. The p-values were interpreted as statistically significant if less than 0.05. “For pathway analysis, a pathway was considered to be significantly enriched if its p-value and Benjamini–Hochberg adjusted p-value were both less than 0.05.”

## Results

### Cell population characterization

Of the 195,718 cells, 102,691 of the cells were SNI condition (52.5%). The SNI cells were composed of 31,852 cells (31.0%) 3-days post-surgery, 35,560 cells (34.6%) 14-days post-surgery, and 35,279 cells (34.4%) 5-months post-surgery. The Sham cells were composed of 27,993 cells (30.1%) 3-days post-surgery, 31,131 cells (33.5%) 14-days post-surgery, and 33,903 cells (36.4%) 5-months post-surgery.

### Analysis of S1R expression by SNI versus Sham condition by time point

S1R expression in all microglia in the SNI condition and Sham condition was compared, across all time points and for each time point separately. From Table 1, there was no evidence of a significant increase in S1R expression in the SNI condition versus Sham condition for the pooled (p-val = 0.081), 3-day post-surgery (p-val = 0.149), 14-day post-surgery (p-val = 0.563), or 5-month post-surgery (p-val = 0.117). However, there is evidence of an increase in S1R expression at both the 3-day (p-val = 1.81E−10) and 14-day (p-val = 6.75E−8) compared to 5-month post-surgery.

**Table 1:** S1R expression of SNI microglia compared to Sham microglia at each timepoint.

Time point	Mean S1R (SNI)	Mean S1R (Sham)	p-val
3 days	0.1577575	0.1544487	0.1488
14 days	0.1544476	0.1549293	0.5634
5 months	0.139183	0.1357059	0.1168
Pooled	0.1502301	0.1477789	0.08073

Timepoints of 3 days, 14 days, and 5 months were separately tested for significant S1R increase in expression, and cells from all timepoints of each condition were combined to test for a significant S1R increase in SNI versus Sham independent of timepoint. There is no statistical evidence of a significant increase in S1R expression in the SNI condition when considering all microglia together.

### Subpopulation analysis of S1R expression by SNI versus Sham condition by time point

**Figure 1:**
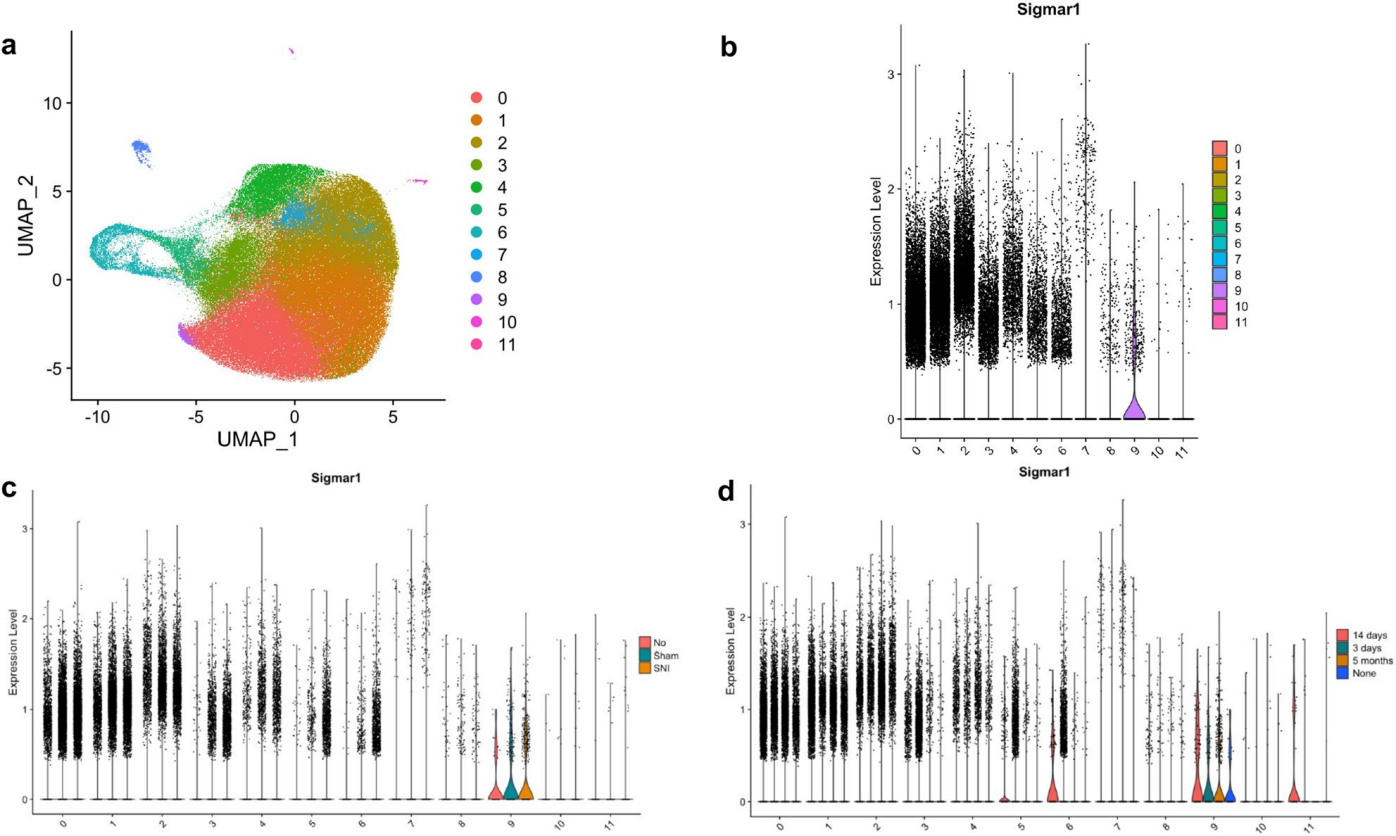
Subpopulation clustering of microglia with cluster number given on the x-axis (**b–d**), (**a**) uniform manifold approximation and planning (UMAP) projection of clustering resulting in 12 distinct subpopulations of microglia, (**b**) S1R expression of each subcluster visualized by violin plot, (**c**) S1R expression of each subcluster split according to experiment condition (SNI, Sham, or None), (**d**) S1R expression of each subcluster split according to experiment timepoint (3 days, 14 days, 5 months, or None). Cells from “None” condition were not included in downstream analyses. S1R likely only has a functional role at levels of expression in cells above a minimal value (i.e., > 0.5). At this “functional threshold” of expression, cluster 7 has a markedly increased distribution of S1R expression. At this high expression, panels c and d show that the microglia are dominated by SNI condition in the 5-month timepoint respectively.

The 12 distinct clusters of microglia subpopulations are visualized in Fig. 1a. An increased frequency of cells was observed in Cluster 7 by Violin Plot (Fig. 1b) at the extremes of S1R expression level (> 1.0). This increased frequency of cells in Cluster 7 at high expression levels for S1R was found to be primarily attributable to SNI and 5-month post-surgery cells (Fig. 1c, d).

Cluster 7 exhibited a significant increase in S1R expression between SNI and Sham conditions for both the pooled (p-val = 0.0037) and 5-month post-surgery (p-val = 0.0064) time points. At the acute 3-day post-surgery time point, Cluster 11 was found to have a significant increase in S1R expression in the SNI condition (p-val = 0.012), but this response did not persist at later time points. Cluster 7 was the only cluster with a significant increase in S1R expression between the two conditions at the 5-month post-surgery time point (Table 2).

**Table 2:** Across all time points, there is only one subcluster (cluster 7) that has significantly increased S1R expression in the SNI condition versus the Sham condition.

Cluster	p-val (pooled)	p-val (3 days)	p-val (14 days)	p-val (5 months)
0	0.7211	0.9781	0.5108	0.07082
1	0.5203	0.9785	0.3721	0.1951
2	0.9048	0.9808	0.3034	0.8202
3	0.5819	0.3615	0.3527	0.9073
4	0.2918	0.6196	0.3174	0.2572
5	0.8458	0.8712	0.5612	0.4412
6	0.1699	0.1705	0.8881	0.266
7	0.003705	0.9176	0.01377	0.006416
8	0.6076	0.6627	0.7881	0.1087
9	0.6606	0.9503	0.5787	0.3409
10	0.2449	0.4477	0.2951	0.2061
11	0.2879	0.01223	0.9132	N/A

Cluster 7 does not have significantly increased expression of S1R at the 3-day timepoint but does at both 14 days and 5 months. At the acute injury time timepoint of 3-days, cluster 11 has significantly increased expression of S1R in the SNI group but does not at 14-days or 5-months.

### Functional analysis of the clusters

**Figure 2:**
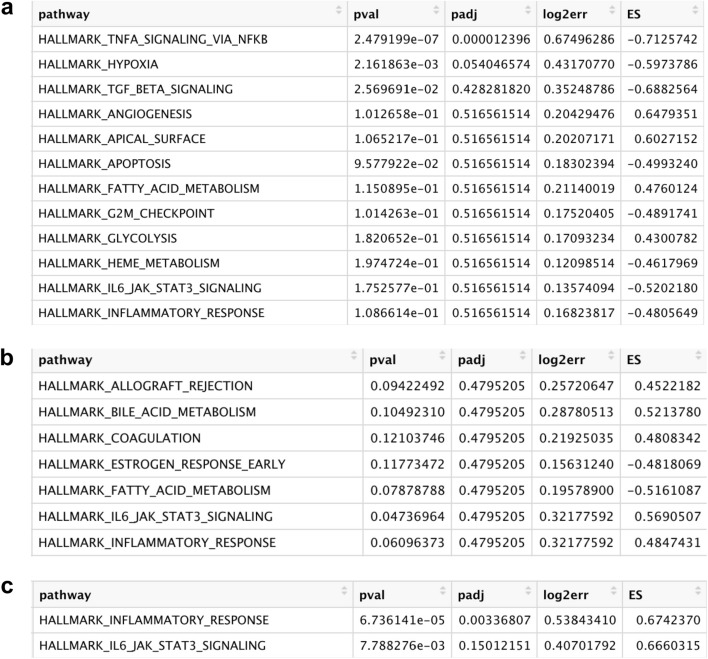
Gene Set Enrichment Analysis (GSEA) pathway analysis results testing hallmark pathway enrichment in SNI cluster 7 microglia compared to Sham cluster 7 microglia at (**a**) 3 days (**b**) 14 days and (**c**) 5 months timepoints. The hallmark inflammatory response pathway is only significant at an adjusted p-value threshold of 0.05 at the 5-month timepoint corresponding to a chronic pain condition. At this timepoint there are no other significant pathways.

To explore the biological role of the identified microglial subpopulation in Cluster 7, pathway enrichment analysis was performed (Fig. 2). At the 3-day post–surgery time point, the only significantly upregulated pathway, in SNI versus Sham conditions, was TNFA Signaling Via NFKB (padj = 0.000012) (Fig. 2a). At the 14-day post-surgery time point, there were no significantly upregulated pathways (Fig. 2b). At the 5-month post-surgery time point, the only significantly upregulated pathway was the inflammatory response pathway (padj = 0.0034) (Fig. 2c).

**Figure 3:**
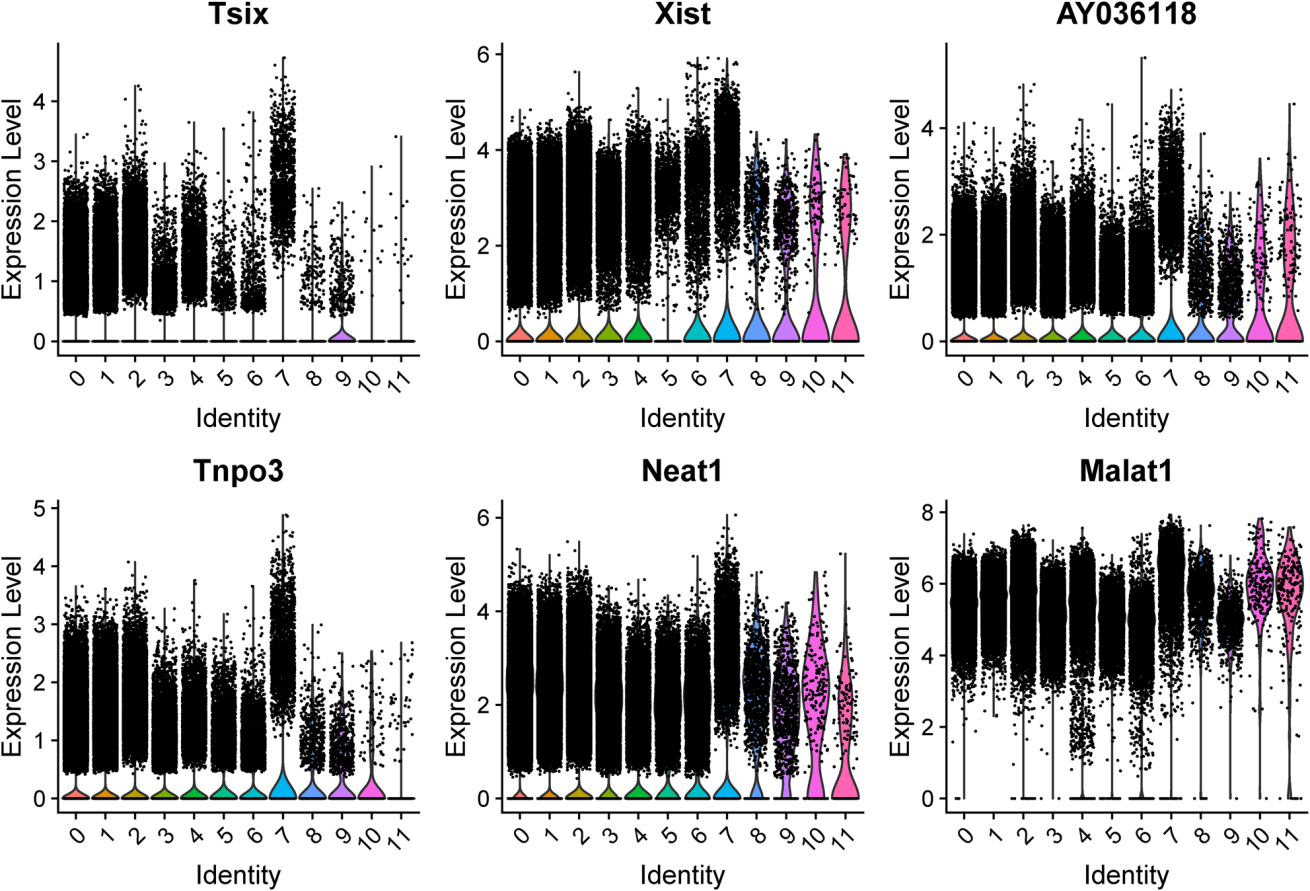
Top candidate marker genes for Cluster 7 microglia across all conditions and timepoints. Top marker genes were selected by degree of fold change in Cluster 7 versus all other clusters. Cluster identity is given on the x-axis.

Marker genes to differentiate Cluster 7 from all other cells at pooled timepoints were determined, with the top six genes by increased fold change (Tsix, Xist, AY036118, Tnpo3, Neat1, Malat1) displayed in Table 3 and violin plots of their expression in Fig. 3.

**Table 3:** The top six marker genes, by average log base twofold change size, of cluster 7 are included.

Marker name	Average Log2 FC	Adjusted p-val
Tsix	1.68	1.37E−135
Xist	1.66	2.99E−235
AY036118	1.56	2.19E−163
Tnpo3	1.49	1.97E−56
Neat1	1.23	0.00E+00
Malat1	1.22	0.00E+00

Their adjusted p-values are all significant and less than 10^–50^. Marker genes refer to the identification of genes that are enriched in cluster 7 microglia cells compared to cells in all other clusters. Data from both sham and SNI conditions are included as both are constituents of Cluster 7 microglia, as well as for the purpose of including active cell function information for the identification of a marker that applies to the cell’s active state. Differentially expressed genes were identified with the Wilcoxon Rank Sum test.

## Discussion

We analyzed the single-cell transcriptomic profiles of L4/5 microglia at the 3-day, 14-day, and 5-month time points after either SNI or Sham surgery to characterize the biological role of S1R in the CNS in response to PNI. This represents the first time that an expressional change in S1R has been studied following SNI in any cell type. A subset of these microglia demonstrated increased expression of S1R following SNI. This microglial subset has a specific functional profile, characterized by an upregulation of the cellular inflammatory response pathway at the 5-month postoperative time point^14^. If the increased expression of S1R in this microglial subpopulation could be identified with imaging modalities such as the S1R radioligand PET/MRI method, it could enable clinicians to distinguish between LBP associated with PNI and LBP due to other causes. This has direct clinical relevance: operative management provides superior outcomes when compared to conservative treatment in cases of LBP with radiculopathy, which is typically caused by spinal nerve compression, a form of PNI. By contrast, fusion and other operative management is no better than intensive nonsurgical rehabilitation in cases of nonradicular LBP^15^. Therefore, identifying PNI by examining the expression of S1R in spinal cord microglia in cases of LBP from an unknown etiology could inform the patient’s decision to pursue operative management.

Increased S1R expression was not seen across all microglia. We hypothesize that this is because only a subset of spinal microglia is involved in the post–injury neuroinflammatory response. Evidence for this is provided in Fig. 1, where S1R is not expressed in most cells within each cluster. However, S1R expression is markedly increased throughout cluster 7. Therefore, cluster 7 microglia could have a unique role in pain or inflammatory signaling. Moreover, S1R expression in cluster 7 microglia is statistically similar across Sham and SNI conditions at the 3-day and 14-day time points, but the cells exposed to SNI show increased expression of S1R at the 5-month time point. This is consistent with the hypothesis that S1R serves as a biomarker of chronic microglial neuroinflammation post–injury. Figure 2 provides additional evidence that the cluster 7 microglial subpopulation has a neuroinflammatory role: GSEA pathway analysis demonstrates that cluster 7 microglia have a significantly upregulated inflammatory response pathway at the 5-month time point post-surgery in the SNI condition relative to the Sham condition. SNI upregulation of the inflammatory pathway is not present at the 3-day or 14-day time points, which is consistent with inflammatory response transitions from acute response to a chronic neuropathic response to nerve injury^16^.

The marker genes to differentiate the identified subpopulation of cluster 7 microglia include Neat1, which further confirms the functional role of the identified subpopulation as Neat1 contributes to neuropathic pain and its downregulation has been shown to inhibit neuroinflammation^17,18^. Neat1^19,20^ and another marker gene for cluster 7 microglia, Malat1^21–23^, likely are members of the axis of diabetic neuropathy pathogenesis, and thus the identified microglial subpopulation may be clinically valuable to distinguish diabetic neuropathic pain from structural sources that may be surgically managed. Therefore, its upregulation in cluster 7 microglia lends further evidence to the hypothesis that this identified subpopulation has a role in promoting neuroinflammation. Thus, evidence from two methods—S1R expression analysis and GSEA pathway analysis—suggest that the identified subpopulation of microglial cells is involved in the pain and inflammatory response secondary to PNI at the five-month post-injury time point.

Given S1R’s potential biological role in the CNS following PNI, S1R expression in spinal cord microglia could be used to differentiate between LBP secondary to PNI and LBP from other etiologies. With the current S1R radiotracer PET/MRI technology, currently S1R expression in the spinal cord has limited clinical interpretations. However, from the results of the current study, higher expression in a specific level of the spinal cord that correlates to symptoms would evidence the identified subpopulation’s response and indication of PNI. However, the potential of insight into pain source using the findings expands with the development of a radiotracer for neuroinflammation. Using one of the marker genes of the identified subcluster, such as the functionally relevant Neat1 or the more specific Tsix/Tnpo3 markers, future studies can translate this to visualize evidence of a chronic PNI response at appropriate levels. With the development of multiplexed PET imaging, expression of this subcluster could be spatially correlated with concurrent S1R expression to identify the subpopulation and PNI functional effect as characterized in this work^24^. Lastly, as PNI often induces symptoms such as allodynia, hypersensitivity and other maps can be combined with S1R expression patterns and cluster markers to localize spinal cord and peripheral injury sites.

### Strengths and limitations

We believe that a major strength of the current work is its high translational actionability. In addition to characterizing a biological role of S1R in the CNS in response to PNI, the results offer a method to differentiate/identify PNI with existing technology—such as the S1R radiotracer used in PET/MRI—that is already in use in clinical trials. If microglia in the spinal canal are activated on the resulting imaging, the pain may be radicular and the level can be correlated with symptoms. This is clinically important as not only is PET/MRI imaging limited in spatial resolution, but there is high overlap between the symptoms of both. Lastly, the controlled nature of the data used to demonstrate S1R upregulation following injury offers high confidence in the results, rather than a clinical study which would have increased uncertainty.

This study has several notable limitations. Sample size is limited although our study has a high level of statistical power. This limitation is common to most transcriptomic study designs in neurosurgery. Second, while the current study used data from animals in a controlled laboratory environment, it is possible that an unidentified confounder could have impacted these findings. Therefore, clinical validation of our results is necessary. A further limitation of the study is that the results are derived from a mouse model of PNI. However, a PNI mouse model has been well established for peripheral neuropathies, and S1R has been extensively studied in rodent populations prior to its translation for human molecular imaging^8,25,26^. Several other critical steps need to take place to validate this method’s ability to differentiate between LBP of PNI and non-PNI etiologies. First, changes in S1R expression in non-neuropathic back pain must be characterized. It is possible that local injury, such as age-related degenerative changes, could cause a similar change in S1R expression, which would limit this approach as a way to differentiate between PNI-related LBP and non-PNI related LBP. Lastly, the SNI induced in the mice that provided data for this study was a tibial and sural nerve ligation. While the tibial and sural nerve are peripheral nerves, it should be confirmed that their ligation produce changes in S1R expression that mirrors changes following radiculopathies at exiting nerve root sites.

## Conclusion

A distinct subcluster of L4/5 microglia was identified which overexpresses S1R following peripheral nerve injury consistent with neuropathic pain inflammatory response functioning. This suggests that an upregulated S1R response in the CNS is a marker for chronic peripheral nerve injury and may be further developed for clinical localization of pain.

## Data Availability

The datasets generated and/or analyzed during the current study are available in the NCBI GEO repository, with accession number GSE162807 at https://www.ncbi.nlm.nih.gov/geo/query/acc.cgi?acc=GSE162807.

## Abbreviations

LBP: Low back pain
S1R: Sigma-1 receptor
CNS: Central nervous system
PNI: Peripheral nerve injury
PET: Positron emission tomography
MRI: Magnetic resonance imaging
SNI: Spared nerve injury
PCA: Principal component analysis
UMAP: Uniform manifold approximation and projection
